# Promoting physical activity: the contribution of regulatory fit

**DOI:** 10.3389/fspor.2025.1564917

**Published:** 2025-05-14

**Authors:** Chao Sun, Yanghui Gao, Jiao Liu, Guoli Zhang, Junhua Dang

**Affiliations:** ^1^ School of Psychology, Beijing Sport University, Beijing, China; ^2^ School of Humanities and Social Sciences, Xi’an Jiaotong University, Xi’an, China; ^3^ Department of Surgical Sciences, Uppsala University, Uppsala, Sweden

**Keywords:** regulatory fit, regulatory focus, exercise, information frame, physical activity

## Abstract

**Background:**

Existing research primarily focuses on external strategies for promoting physical activity, while the influence of individual characteristics on engagement in physical activities has been largely overlooked. This study aims to explore the impact of combining individual regulatory focus and exercise information frame on cognition, emotion, intention, and behavior based on the regulatory fit theory.

**Methods:**

In Study 1, participants were randomly assigned to read one of two types of exercise information with different frames but the same content after measuring their chronic regulatory focus. Subsequently, they completed a measure of exercise-related cognition, emotion, and intention. Study 2 replicated this by manipulating situational regulatory focus and extended this by tracking participants' actual engagement in physical activity for one week.

**Results:**

Both studies revealed significant interactions between regulatory focus and the information frame on information value, emotional intensity, and behavioral intention, indicating the regulatory fit effect. More importantly, Study 2 found a significant interaction on vigorous physical activity during one week after the manipulation of situational focus.

**Conclusion:**

When chronic/situational regulatory focus fit with the exercise information frame, individuals evaluated the exercise information more positively, showed more positive emotions, were more willing to take part in exercise, and engaged in more actual physical activity.

## 1 Introduction

According to international guidelines, adults are advised to engage in 150–300 min of moderate-intensity or 75–150 min of vigorous-intensity physical activity per week, or a combination of both, in order to meet the recommended aerobic exercise guidelines (1). In fact, 27.5% of adults and 81% of adolescents fail to engage in sufficient physical activity (1). The prevalence of insufficient physical activity among Chinese adults exhibited an increasing trend from 2010 to 2018, with over one-fifth of adults failing to meet the threshold (2). Physical inactivity represents a significant global public health concern and ranks as the fourth leading cause of mortality (3). Mounting evidence has established a significant association between habitual physical activity and multidimensional neurophysiological adaptations, encompassing neurobiological homeostasis maintenance, augmented emotional regulation capacity, optimized cognitive processing efficiency, functional reorganization of mesolimbic reward pathways (4), circadian entrainment-mediated sleep quality enhancement, hypothalamic-pituitary-adrenal axis modulation governing cortisol dynamics (5), and prosocial behavioral facilitation through interpersonal neurobiological mechanisms (6). Conversely, insufficient physical activity elevates risks for non-communicable diseases spanning hypertension, type II diabetes mellitus, cardiovascular pathologies, breast/colorectal malignancies, and depressive disorders (7). The prevalence of insufficient physical activity is significantly high in several countries, with rates of 40% in the United States, 34% in India, 47% in Brazil, and 42% in New Zealand being reported (8). Fortunately, physical activity is a modifiable healthy behavior (9). However, existing research primarily focuses on external strategies such as financial incentives (10), social marketing (11), wearable devices (3), and smartphone apps (12) to promote physical activity. Current research has inadequately addressed the interaction effects between individual characteristics and externally-driven intervention strategies. Emerging evidence suggests that congruence between participants' regulatory focus and externally-framed informational stimuli demonstrates substantial influence on health behavior engagement, including but not limited to: adoption rates of smartwatch-based health applications (13), adherence to nutritional guidelines (14), and consumption patterns of athletic merchandise (15). Consequently, it is crucial from both theoretical and practical perspectives to investigate how to deliver appropriate and tailored information to participants based on their regulatory focus characteristics in order to enhance their adoption of physical activity. This paper investigates the impact of combining individual regulatory focus and exercise information on cognition, emotion, intention, and behavior based on the regulatory fit theory. Specifically, it addresses the following questions: Does the regulatory fit between trait focus and information statement influence individuals' perceived value and willingness to engage in exercise? What are the effects of priming individuals with different state regulatory focuses on cognition, emotion, intention, and behavior? Is there consistency between trait regulatory fit effect and state regulatory fit effect in the domain of exercise?

### 1.1 Regulatory fit theory

In recent years, the regulatory fit theory has increasingly been applied in the realm of health behavior (13, 16, 17). This theory is rooted in regulatory focus and offers various strategies or information to enhance individuals' awareness of both “gain” and “loss”. Regulatory focus refers to two motivational tendencies that individuals exhibit in the process of goal realization: promotion focus and prevention focus (18). Promotion-focused individuals are concerned with achieving ideal goals, especially whether they can progress, grow, and achieve positive results. In contrast, those with a prevention focus are based on the ought goals and pay close attention to preventing harm and avoiding adverse outcomes (19). Regulatory focus involves two different types: chronic focus and situational focus. The former is a personality tendency determined by an individual's growth environment as well as their experience of success or failure, whereas the latter is a temporary motivation orientation induced by the current task or situation (15).

The conventional perspective, which is based on outcome value, posits that conduct assessment depends on the discrepancy between its benefits and costs—in other words, its result value. A positive result value indicates appropriate behavior. Advances in research have led scholars to discover the process value assessment framework. Building upon this foundation, Higgins proposed the regulatory fit theory (20). The theory of regulatory fit emphasizes the interplay between an individual's regulatory focus and their behavioral strategy in goal pursuit and decision-making. Specifically, a promotion-focused individual adopts a desirability-proximity approach, while a prevention-focused individual employs a vigilance-avoidance strategy. The alignment creates a sense of “rightness” or regulatory fit, which subsequently influences individuals' behavioral cognition. Firstly, regulatory fit has the potential to enhance individuals' perceived value. In Higgins' mug auction experiment, participants who adopted a regulatory focus-consistent strategy exhibited an increase in their monetary valuations of target objects (e.g., coffee mugs). Moreover, the value-enhancement effect induced by regulatory fit extends beyond the immediate context of the original goal-directed behavior (such as canine friendliness ratings), thereby demonstrating a cross-situational generalization (21). Moreover, regulatory fit enhances individuals' experience of heightened emotional valence and strengthens their behavioral intentions. Pfeffer's study revealed that participants with a prevention focus exhibited greater behavioral intention following exposure to negative messages, while those with a promotion focus reported more positive emotions both retrospectively and prospectively after reading positive messages (22). Thirdly, regulatory fit functions as a motivational mechanism driving individuals' engagement in advocacy behaviors, indicating that congruence between an individual's regulatory focus and the strategic framing of task objectives enhances their propensity for goal-congruent proactive engagement. Empirical evidence suggests that when the information frame aligns with the induced situational regulatory focus, sedentary and less active individuals exhibit increased participation in sit-ups, squats, planks, and wall-sits (23). In conclusion, regulatory focus operates in conjunction with behavioral strategies or information frames to produce effects. When there is a fit between regulatory focus and the chosen strategy/frame, individuals' cognition, motivation, and behavior are more likely to be influenced (24).

### 1.2 The interaction between the regulatory focus and frame effect

The extant literature suggests that regulatory fit and frame effect theories are significant factors in influencing persuasion, behavioral motivation, and decision preference. The framing effect constitutes a cognitive bias wherein equivalent objective information, when differentially contextualized through representational variations (i.e., framing), systematically alters decision outcomes and value assessments in judgmental contexts (25). It is typically divided into the positive frame, which emphasizes the advantages of adopting the behavior, and the negative frame, which focuses on the losses caused by not implementing the behavior (26). According to the regulatory fit theory (27), the promotion-focused individuals are more sensitive to the positive frame, while those with a prevention-focus are more inclined to accept the negative frame. Higgins postulated that the interaction between trait regulatory focus and information presentation would enhance the persuasive impact of information (13). Wang's study demonstrated that aligning consumers' focus with the advertising information framework can effectively activate their attitudes and purchase intentions (28).

In the field of exercise psychology, only a few studies have explored the effect of regulatory fit on exercise motivation and behavior (29). For example, Pfeffer explored the regulatory fit effect between chronic regulatory focus and the exercise information frame and found that participants with a preventive focus reported stronger behavioral intention after reading the negative message while those with a promotion focus reported more retrospective and prospective positive emotions after reading the positive message (18). Latimer et al. found messages that fitted individuals' regulatory focus led to greater physical activity participation and more positive feelings than non-fit messages (30).

### 1.3 Current research

Despite compelling evidence for regulatory fit's role in exercise motivation, three critical limitations hinder theoretical refinement and practical implementation. First, the predominant focus on chronic regulatory fit—aligning stable promotion/prevention traits with message framing—fails to address whether situational priming of transient motivational states can produce comparable or amplified effects, leaving the trait-state interplay in exercise behavior unresolved. Second, while laboratory studies demonstrate acute regulatory fit effects on cognitive and intentional outcomes (22, 30), their ecological validity remains questionable given the lack of behavioral tracking in real-world contexts. For instance, Kay and Grimm's (23) single-session paradigm with exercise-naïve individuals cannot confirm effect persistence beyond controlled settings. Third, previous studies have often dichotomized exercise experience into “naïve” and “highly experienced” categories (23), thereby overlooking individuals with moderate exercise engagement—one of the most prevalent yet understudied group in non-athletic populations. The current research focuses on regulatory fit mechanisms within this specific group.

To resolve these interconnected limitations, we conduct a sequential investigation targeting moderate exercisers—a sport university undergraduate cohort representing non-specialized populations. Study 1 establishes baseline effects by replicating chronic regulatory fit's immediate impacts on exercise cognition and intention. Study 2 innovates through dual methodologies: (a) experimental induction of situational regulatory focus to disentangle state-level effects from chronic traits, and (b) seven-day ecological assessment tracking real-world exercise frequency, duration, and intensity. This design uniquely bridges laboratory precision with ecological validity while testing two critical hypotheses: (1) whether transient motivational states can override habitual trait-based responses, and (2) how regulatory fit mechanisms operate in individuals with established (but non-expert) exercise routines.

## 2 Study 1: regulatory fit effect between chronic regulatory focus and the information frame

Study 1 aims to conceptually replicate previous studies investigating the immediate effect of regulatory fit between chronic regulatory focus and the exercise information frame on exercise-related cognition, emotion, and intention (18). We expect the exercise information frame that is congruent with one's chronic regulatory focus would bring about higher information value, more positive emotional experience, and more willingness to exercise.

### 2.1 Methods

#### 2.1.1 Experimental design and procedures

The experimental design incorporated both categorical and continuous independent variables. Information framing (positive/negative) was manipulated as a dichotomous factor, while chronic regulatory focus was measured as a continuous construct. First, participants were instructed to complete the regulatory focus questionnaire. Second, participants received the manipulation of the exercise information frame. They were randomly assigned to read one of two pieces of information with different frames but the same theme and content. Finally, the dependent variables were measured by the persuasive effect questionnaire that taps exercise-related cognition, emotion, and intention.

#### 2.1.2 Participants

We conducted *a priori* power analysis using G*Power 3.1.9.7 to estimate the required sample size (31). Based on effect sizes from regulatory fit studies in exercise contexts [ (32): *f* = 0.34–0.39; (33): *f* = 0.34–0.36; (23): *f* = 0.23–0.42], we conservatively assumed a medium effect size (*f* = 0.30). For a 2 × 2 factorial ANOVA examining the interaction between regulatory focus and information framing, we set *α* = 0.05, power (1 − *β*) = 0.80, and *f* = 0.30. The analysis indicated a target sample of 90. The final cohort comprised 121 non-athlete undergraduate students (48 male, 73 female; *M*_age = 22.91 years, *SD* = 3.22) recruited from a sports university, representing a population characterized by moderate but non-elite physical activity engagement. As detailed in Table 1, baseline assessments confirmed participants' weekly vigorous-intensity, moderate-intensity, and walking activity levels corresponded to normative values for young adults established in global population studies (1), effectively controlling for potential athletic performance confounds.

**Table 1:** Baseline information of participants in Study 1 (*M* ± *SD*).

Dependent variable	Information frame	Chronic regulatory focus		*F*	*p*
		Promotion focus	Prevention focus		
Age (years)	PF	20.40 ± 1.71	20.66 ± 1.60	0.17	0.67
	NF	20.32 ± 1.67	20.32 ± 1.81		
Height (cm)	PF	173.17 ± 8.70	171.80 ± 8.44	0.62	0.43
	NF	170.52 ± 7.73	171.80 ± 7.33		
Weight (kg)	PF	55.08 ± 7.80	55.73 ± 7.05	0.50	0.47
	NF	56.12 ± 7.93	54.80 ± 7.26		
BMI (kg/m²)	PF	21.11 ± 3.17	21.63 ± 4.03	0.76	0.38
	NF	20.16 ± 3.11	19.58 ± 3.26		
Walking PA	PF	434.48 ± 100.59	433.20 ± 103.06	0.64	0.42
	NF	450.68 ± 108.26	420.16 ± 86.09		
Moderate PA	PF	939.28 ± 167.11	901.00 ± 145.15	1.56	0.21
	NF	897.48 ± 142.73	934.12 ± 190.61		
Vigorous PA	PF	947.08 ± 203.53	986.20 ± 184.05	0.13	0.71
	NF	882.96 ± 195.04	948.00 ± 184.05		
Total PA	PF	2,320.85 ± 278.67	2,320.40 ± 220.49	0.55	0.45
	NF	2,231.12 ± 273.78	2,302.29 ± 271.61		

PF, positive frame; NF, negative frame; PA, physical activity.

#### 2.1.3 Manipulation of the information frame

We created two pieces of exercise information with different frames but the same content. The positive frame emphasizes positive results (i.e., “Physical activity can raise metabolism, improve physique, boost immunity, and decrease disease incidence”), while the negative frame stresses negative results (i.e., “Absence of physical activity can reduce metabolism, negatively affect body image, diminish immunity, and increase disease incidence”).

#### 2.1.4 Measures

##### 2.1.4.1 Regulatory focus questionnaire

The Chinese version of the regulatory focus questionnaire (see Supplementary Materials), which has six questions on the promotion focus dimension and four on the prevention focus dimension, was translated and revised by Yao et al. (34). Eight of the questions are scored on a scale ranging from 1 (*never*) to 5 (*always*), while the other two are scored ranging from 1 (*completely wrong*) to 5 (*completely right*). To capture individuals' dominant regulatory focus, a difference score was calculated by subtracting the prevention focus score from the promotion focus score. This continuous variable reflects the relative strength of regulatory focus, with higher scores indicating a promotion-oriented focus and lower scores indicating a prevention-oriented focus. In this study, Cronbach's *α* of the promotion focus dimension and the prevention focus dimension are 0.68 and 0.70, respectively.

##### 2.1.4.2 Persuasive effect questionnaire (PEQ)

The questionnaire was constructed based on three theoretically grounded dimensions—cognitive appraisal, affective response, and behavioral intention—derived from regulatory fit theory and persuasion research (13, 18). These dimensions were operationalized as follows: (1) information value, reflecting participants' evaluations of the perceived validity, credibility, and persuasive efficacy of exercise-related content; (2) emotional intensity, quantifying the magnitude of both positive (e.g., pleasure) and negative (e.g., sadness) affective states evoked by the information; (3) behavioral intention, assessing participants' motivation to engage with exercise recommendations and their commitment to future behavioral modifications (see Supplementary Materials). All items employed a 5-point Likert-type scale (1 = Extremely inconsistent to 5 = Extremely consistent). Confirmatory factor analysis validated the hypothesized three-factor structure (*χ*²/df = 1.18; CFI = 0.99; TLI = 0.99; RMSEA = 0.04; SRMR = 0.05), with psychometric analyses confirming reliability at both subscale (information value: *α* = 0.78; emotional intensity: *α* = 0.72; behavioral intention: *α* = 0.85) and the full-scale level (*α* = 0.82), exceeding conventional thresholds for measurement precision.

##### 2.1.4.3 International physical activity questionnaire (IPAQ)

This questionnaire asked participants to report how much vigorous physical activity, moderate physical activity, and walking physical activity they have done during the last 7 days. The amount of each type of physical activity was calculated as follows: The vigorous physical activity score = 8 × days of vigorous activity × time of vigorous activity per day; the moderate physical activity score = 4 × days of moderate physical activity × time of moderate physical activity per day; the walking physical activity score = 3.3 × walking days × walking time per day; the total physical activity score = the vigorous physical activity score + the moderate physical activity score + the walking physical activity score (35).

### 2.2 Results

#### 2.2.1 Preliminary analyses

The means and standard deviations (*M* ± *SD*) for the negative and positive frames across each variable were as follows: chronic regulatory focus (0.41 ± 0.53 vs. 0.50 ± 0.49), information value (5.23 ± 0.97 vs. 5.08 ± 0.85), emotional intensity (4.93 ± 0.96 vs. 4.58 ± 0.92), behavioral intention (5.21 ± 1.23 vs. 4.91 ± 0.91), with no statistically significant between-group differences observed (all *p*s > 0.05).

**Figure 1:**
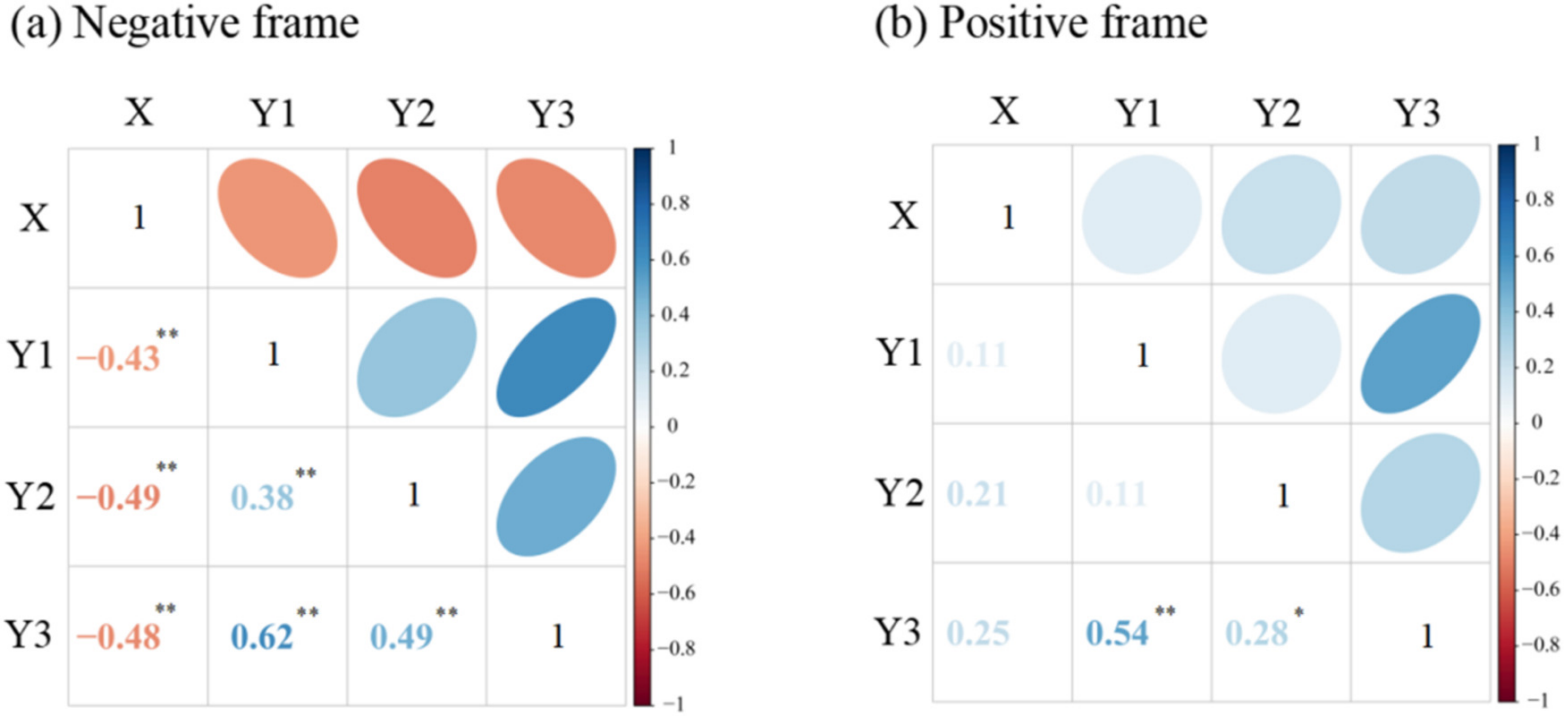
The correlation matrix among variables. **(a)** Negative frame, **(b)** Positive frame. X, chronic regulatory focus, Y1, information value, Y2, emotional intensity, Y3, behavioral intention. The lower triangular section of the matrix presents specific correlation coefficient values, whereas the upper triangular section illustrates the correlations' direction and strength via the ellipses' color and shape. Blue ellipses signify positive correlations, and red ellipses indicate negative ones. A darker or more flattened ellipse denotes a stronger correlation.

Figure 1 presents the bivariate correlation matrices for both framing conditions. Within the negative framing condition, chronic regulatory focus demonstrated significant inverse associations with information value (*r* = −0.43, *p* < 0.01), emotional intensity (*r* = −0.49, *p* < 0.01), and behavioral intention (*r* = −0.48, *p* < 0.01). Positive correlations emerged between information value and emotional intensity (*r* = 0.38, *p* < 0.01), as well as behavioral intention (*r* = 0.62, *p* < 0.01). Emotional intensity further correlated positively with behavioral intention (*r* = 0.49, *p* < 0.01). Under positive framing, behavioral intention exhibited positive associations with both information value (*r* = 0.54, *p* < 0.01) and emotional intensity (*r* = 0.28, *p* < 0.01).

#### 2.2.2 Regulatory fit effect between chronic regulatory focus and the information frame

Given that chronic regulatory focus (a continuous independent variable) and exercise information frame (a dichotomous independent variable) jointly influence persuasion outcomes (continuous dependent variables), we employed Hayes' PROCESS macro (Model 1) to examine their interactive effects, following established analytical procedures for moderation analysis. As shown in Table 2, chronic regulatory focus significantly predicted information value (*β* = −0.34, *p* < 0.05) and emotional intensity (*β* = −0.31, *p* < 0.05). The exercise information frame also directly predicted emotional intensity (*β* = −0.33, *p* < 0.05). Notably, the interaction between chronic regulatory focus and exercise information frame significantly predicted information value (*β* = 0.99, *p* < 0.01), emotional intensity (*β* = 1.31, *p* < 0.01), and behavioral intention (*β* = 1.38, *p* < 0.01).

**Table 2:** Moderating model of information frame between chronic regulatory focus and persuasive effect.

Dependent variance	Independent variance	*R* ^2^	*F*	*β*	*SE*	*t*
Information value		0.13	5.69**			
	X			−0.34	0.15	−2.20*
	M_O_			−0.12	0.16	−0.77
	X*M_O_			0.99	0.31	3.20**
Emotional intensity		0.19	9.30**			
	X			−0.31	0.15	−2.00*
	M_O_			−0.33	0.15	−2.08*
	X*M_O_			1.31	0.31	4.20**
Behavioral intention		0.15	6.77**			
	X			−0.29	0.18	−1.61
	M_O_			−0.28	0.18	−1.48
	X*M_O_			1.38	0.36	3.77**

X, chronic regulatory focus; M_O_, exercise information frame.

* *p* < 0.05.

** *p* < 0.01.

**Figure 2:**
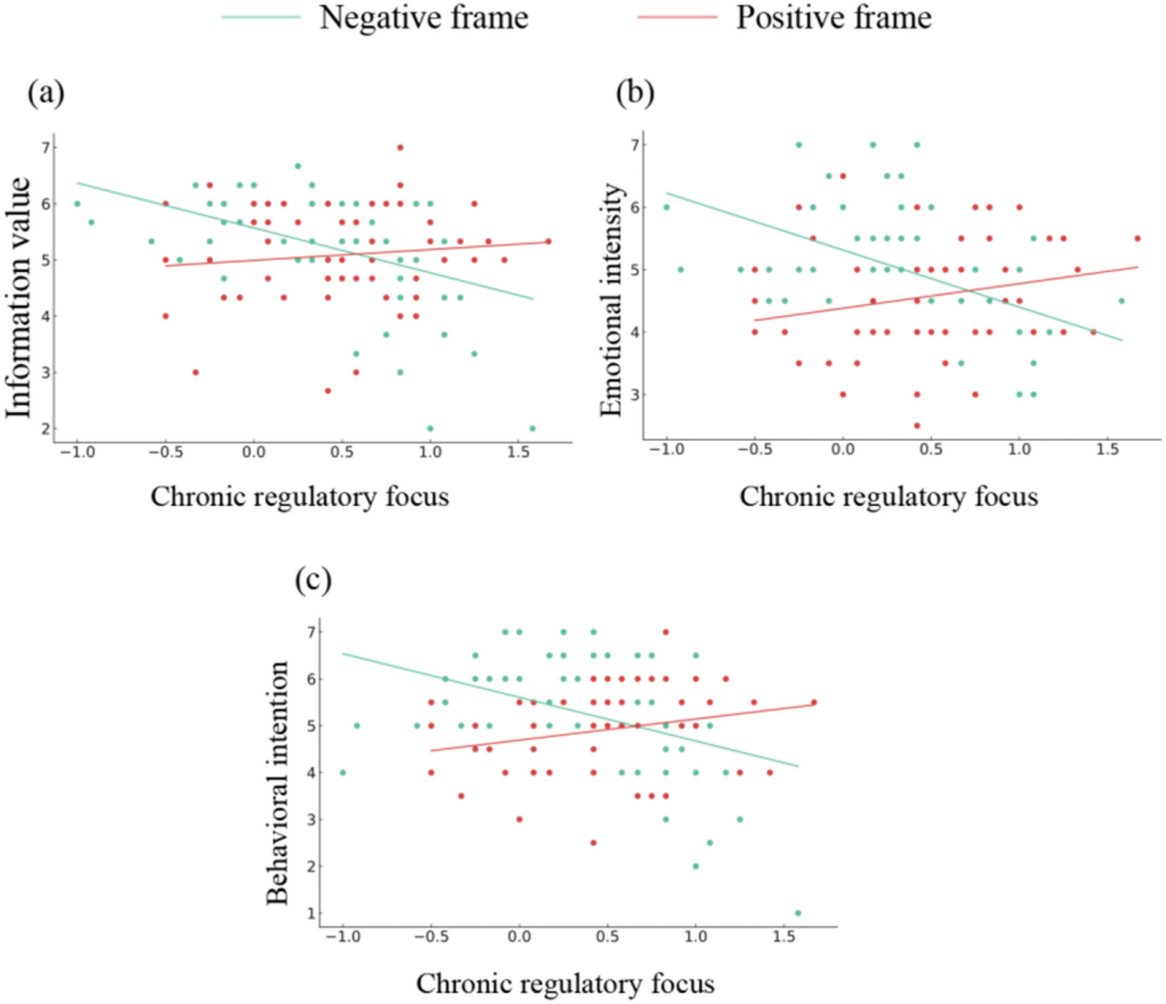
Interaction of chronic regulatory focus and exercise information frame predicting regulatory fit effect in Study 1. **(a)** Information value, **(b)** Emotional intensity, **(c)** Behavioral intention.

Subsequent simple slope analyses demonstrated that chronic regulatory focus exerted significant adverse effects on information value [effect = −0.80, 95% CI (−1.20, −0.39)], emotional intensity [effect = −0.91, 95% CI (−1.31, −0.51)], and behavioral intention [effect = −0.93, 95% CI (−1.41, −0.46)] within the negative framing condition. Notably, these effects failed to attain statistical significance under positive framing conditions. Specifically, prevention-focused participants displayed elevated scores for information value, emotional intensity, and behavioral intention following exposure to negative framing stimuli. Conversely, promotion-focused individuals exhibited comparatively higher scores on these dependent variables when presented with positive framing, though these associations failed to reach statistical significance. The corresponding results are graphically depicted in Figure 2.

### 2.3 Discussion

Study 1 found that regulatory fit of chronic regulatory focus and the exercise information frame led to higher evaluation of exercise information, more positive emotional experience, and more willingness to exercise. However, it has been argued that behavioral intention might not always transfer to actual behavior, especially in the exercise domain (19). Therefore, in Study 2, in addition to testing the immediate effect of regulatory fit on information value, emotional intensity, and behavioral intention, we also tracked participants' actual exercise behavior in one week to examine the longitudinal effect of regulatory fit. We also used a dual task to prime participants' situational regulatory focus to explore whether it has a similar effect as chronic regulatory focus.

## 3 Study 2: regulatory fit effect of situational regulatory focus and the exercise information frame

### 3.1 Methods

#### 3.1.1 Experimental design and procedures

**Figure 3:**
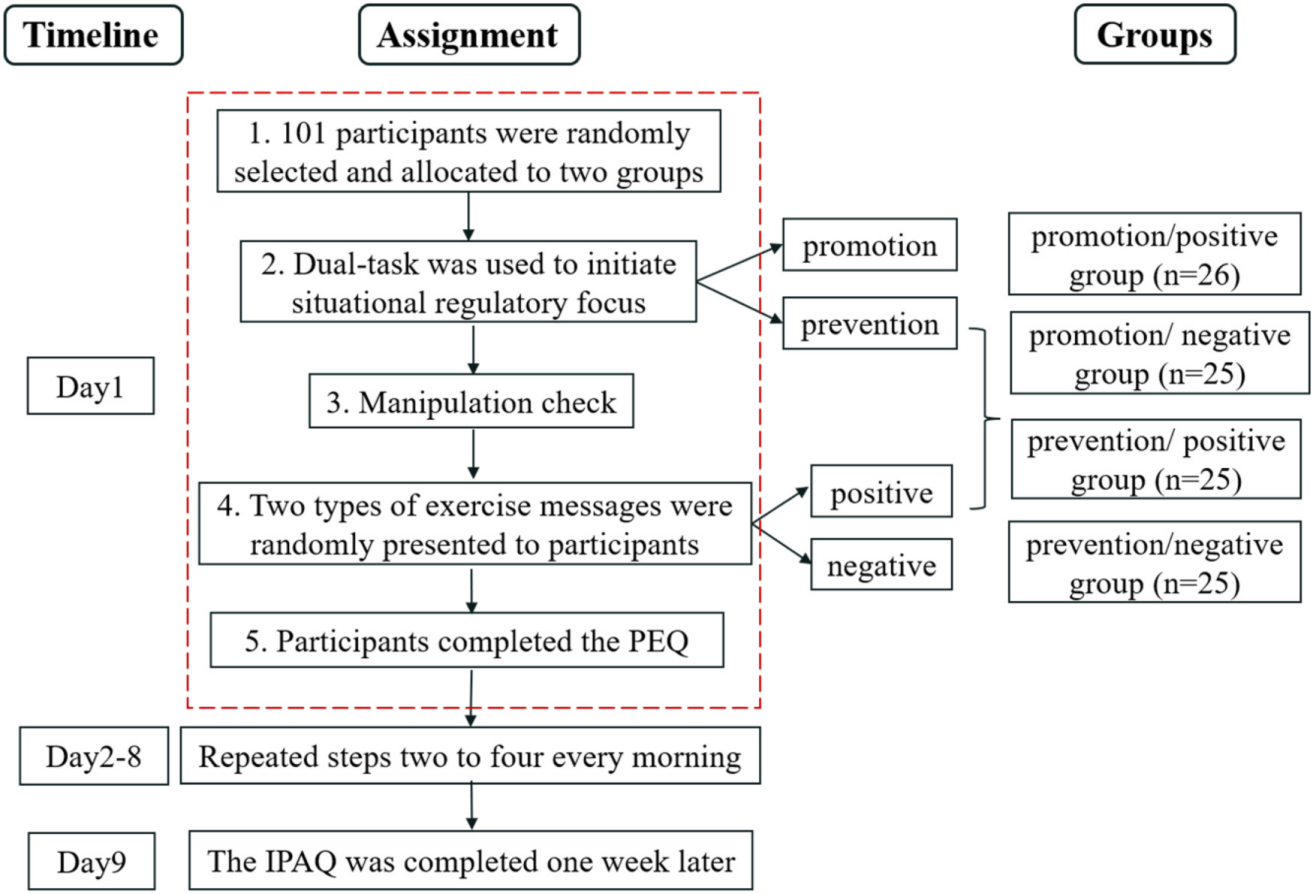
The experimental procedures of Study 2. PEQ, persuasive effect questionnaire; IPAQ, International Physical Activity Questionnaire.

We adopted a 2 (situational regulatory focus: promotion vs. prevention) × 2 (exercise information frame: positive vs. negative) between-subjects design. On day 1, participants were first randomly assigned to be primed by either a promotion focus or a prevention focus. After the manipulation check questionnaire, they received the same manipulation of the exercise information frame shown in Study 1. Finally, they completed the persuasive effect questionnaire that taps exercise-related cognition, emotion, and intention, as shown in Study 1. During day 2 to day 8, they repeated steps two to four every morning in the same manner as on the initial day. On day 9, they were invited to fulfil the IPAQ. The experimental procedure was depicted in Figure 3.

#### 3.1.2 Participants

As in Study 1, *a priori* power analysis using G*Power 3.1.9.7 for a 2 × 2 factorial design (*α* = 0.05, power = 0.80, *f* = 0.30) indicated a target sample size of 90. We recruited 101 college students (32 males, 69 females; *M*_age = 21.23, *SD* = 2.81) from the same university. As presented in Table 3, baseline demographic profiles confirmed participants' weekly vigorous-intensity, moderate-intensity, and walking activity levels corresponded to normative ranges for young adults documented in global epidemiological research (1), thereby maintaining population representativeness while controlling for athletic performance biases.

**Table 3:** Baseline information of participants in Study 2 (*M* ± *SD*).

Dependent variable	Information frame	Situational regulatory focus		*F*	*p*
		Promotion focus	Prevention focus		
Age (years)	PF	20.36 ± 1.77	21.10 ± 1.58	1.63	0.20
	NF	20.62 ± 1.68	20.52 ± 1.75		
Height (cm)	PF	171.03 ± 8.29	170.40 ± 8.90	1.40	0.23
	NF	171.20 ± 8.68	174.48 ± 8.31		
Weight (kg)	PF	55.43 ± 10.14	53.10 ± 10.47	1.50	0.22
	NF	54.70 ± 10.76	51.44 ± 10.41		
BMI (kg/m²)	PF	20.20 ± 3.54	20.40 ± 3.46	0.01	0.90
	NF	20.00 ± 2.97	20.04 ± 3.24		
Walking PA	PF	424.66 ± 95.03	447.16 ± 86.27	1.38	0.24
	NF	440.37 ± 96.24	420.68 ± 95.47		
Moderate PA	PF	893.13 ± 162.91	914.26 ± 147.74	1.18	0.27
	NF	990.83 ± 154.00	945.08 ± 172.82		
Vigorous PA	PF	913.36 ± 160.12	927.43 ± 181.26	0.95	0.33
	NF	935.50 ± 162.09	1,014.40 ± 185.12		
Total PA	PF	2,231.16 ± 248.41	2,288.86 ± 234.54	0.18	0.66
	NF	2,366.70 ± 262.25	2,380.16 ± 325.31		

PF, positive frame; NF, negative frame; PA, physical activity.

#### 3.1.3 Manipulation of situational regulatory focus

The Dual-Task paradigm was employed to initiate the situational regulatory focus (36). The first one was a self-directed task, and the second one was a maze task on paper. In order to prime the promotion focus, participants were instructed to recollect their past or recent hopes or wishes and then complete the maze task. Specifically, there was a rat in the middle of the maze and a piece of cheese at the exit, and participants had to help the rat find the right path to get the cheese. To induce the prevention focus, individuals were required to recall their past or recent responsibilities or obligations and then fulfil the maze task. Specifically, participants needed to guide the rat to find the right path to escape the maze wherein a hungry eagle prowled over it and threatened the rat.

#### 3.1.4 Manipulation of the information frame

The information frame manipulation was identical to that used in Study 1.

#### 3.1.5 Measures

As Study 1, we used the PEQ to measure the persuasive effect and the IPAQ to measure physical activity. To do the manipulation check of regulatory focus priming, we used the questionnaire of Pham and Avnet (37) (see Supplementary Materials). It is composed of 3 questions that scored 7 points, with each having two opposite choices (e.g., I prefer to do whatever I promise to do vs. I prefer to go wherever I want to go). Individuals with lower scores are more inclined to be the prevention focus, while those with higher score are more likely to be the promotion focus.

### 3.2 Result

#### 3.2.1 Manipulation of situational regulatory focus

The *t* test was used to check the manipulation of situational regulatory focus, which showed a significant difference between the two groups, *M*_promotion focus grou*p*_ = 4.69; *M*_prevention focus grou*p*_ = 4.14, *t* (99) = 3.02, *p* < 0.01, indicating that the manipulation of situational regulatory focus was successful.

#### 3.2.2 Regulatory fit effect of situational regulatory focus and the exercise information frame

##### 3.2.2.1 Preliminary analyses

As shown in Table 4, in the positive frame condition, participants primed with a promotion focus scored higher on information value, emotional intensity, behavioral intention, and physical activity measured by the international physical activity questionnaire than those primed with a prevention focus. In the negative frame condition, the results were just the opposite. We conducted ANOVAs to test the significance, taking situational regulatory focus and the exercise information frame as independent variables and information value, emotional intensity, behavioral intention, and physical activity measured by the international physical activity questionnaire as dependent variables.

**Table 4:** Description statistics of variables in Study 2 (*M* ± *SD*).

Dependent variable	Information frame	Situational regulatory focus	
		Promotion focus	Prevention focus
Information value	Positive frame	5.69 ± 0.51	4.81 ± 0.87
	Negative frame	4.94 ± 0.89	5.17 ± 0.73
Emotional intensity	Positive frame	5.42 ± 0.99	4.46 ± 0.93
	Negative frame	4.00 ± 0.93	4.64 ± 0.74
Behavioral intention	Positive frame	5.33 ± 1.00	4.16 ± 0.84
	Negative frame	4.86 ± 0.90	5.08 ± 0.97
Walking PA	Positive frame	393.85 ± 264.14	363.80 ± 262.75
	Negative frame	277.20 ± 163.38	411.60 ± 306.55
Moderate PA	Positive frame	951.54 ± 627.23	728.00 ± 347.95
	Negative frame	692.80 ± 467.54	823.20 ± 551.41
Vigorous PA	Positive frame	2,729.23 ± 1,083.12	1,190.40 ± 424.15
	Negative frame	819.20 ± 519.12	1,737.60 ± 866.11
Total PA	Positive frame	4,074.61 ± 1,440.93	2,282.20 ± 579.47
	Negative frame	1,789.20 ± 797.87	2,972.40 ± 1,635.57

PA, physical activity.

##### 3.2.2.2 Combined effects of situational regulatory focus and message frame on persuasive outcomes and actual physical activity

**Figure 4:**
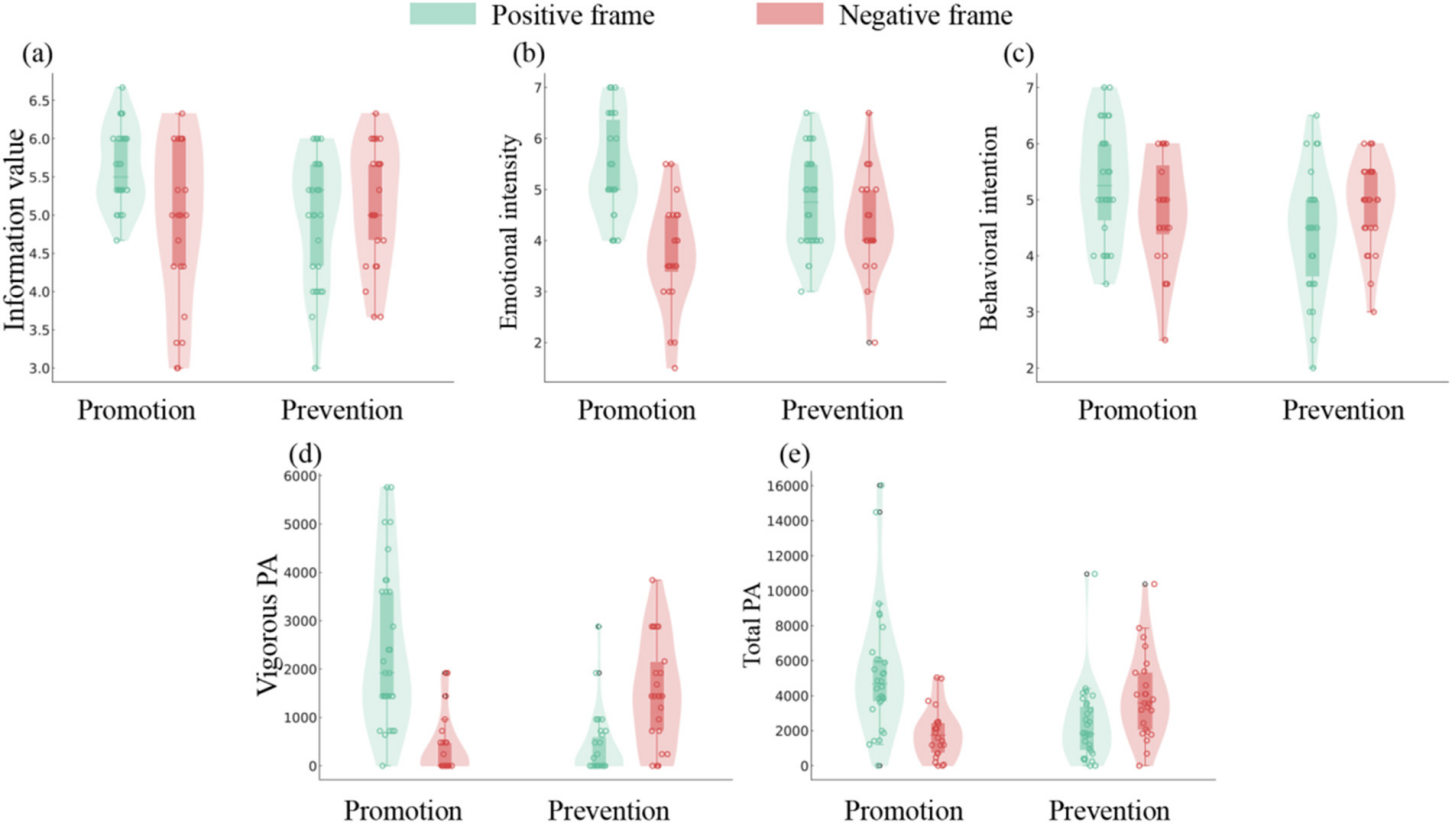
Interaction of situational regulatory focus and exercise information frame predicting regulatory fit effect in Study 2. **(a)** Information value, **(b)** Emotional intensity, **(c)** Behavioral intention, **(d)** Vigorous physical activity, **(e)** Total physical activity. PA, physical activity.

(1) Information value: The main effect of situational regulatory focus was significant, *F*(1, 97) = 4.58, *p* = 0.04, *η_p_*^2^ *=* 0.05. However, the main effect of the exercise information frame was not significant, *F*(1, 97) = 1.60, *p* = 0.21. Importantly, there was a significant interaction between situational regulatory focus and the exercise information frame (see Figure 4A), *F*(1, 97) = 13.16, *p* < 0.01, *η_p_*^2^ *=* 0.12.
(2) Emotional intensity: The main effect of situational regulatory focus was not significant, *F*(1, 97) = 0.80, *p* = 0.37, while the main effect of the exercise information frame was significant, *F*(1, 97) = 11.81, *p* < 0.01, *η_p_*^2^ *=* 0.11. Furthermore, the interaction between situational regulatory focus and the exercise information frame was significant (see Figure 4B), *F*(1, 97) = 19.65, *p* < 0.01, *η_p_*^2^ *=* 0.17.
(3) Behavioral intention: The main effect of the exercise information frame was not significant, *F*(1, 97) = 1.75, *p* = 0.19, whereas the main effect of situational regulatory focus [*F*(1, 97) = 7.63, *p* = 0.01, *η_p_*^2^ *=* 0.07] and the interaction [*F*(1, 97) = 16.37, *p* < 0.01, *η_p_*^2^ *=* 0.14] were significant (see Figure 4C).
(4) Vigorous physical activity: The main effect of situational regulatory focus [*F*(1, 97) = 4.05, *p* = 0.04, *η_p_*^2^ *=* 0.04] and the exercise information frame [*F*(1, 97) = 19.56, *p* < 0.01, *η_p_*^2^ *=* 0.17] were significant. Moreover, the interaction was also significant (see Figure 4D), *F*(1, 97) = 19.65, *p* < 0.01, *η_p_*^2^ *=* 0.17.
(5) Moderate physical activity: Both the main effect [*F*(1, 97)_regulatory focus_ = 1.06, *p* = 0.31, *F*(1, 97)_information frame_ = 0.65, *p* = 0.42] and the interaction [*F*(1, 97) = 3.03, *p* = 0.09] were not significant.
(6) Walking physical activity: Neither the main effect [*F*(1, 97)_regulatory focus_ = 1.79, *p* = 0.18, *F*(1, 97) _information frame_ = 0.46, *p* = 0.05] nor the interaction [*F*(1, 97) = 2.63, *p* = 0.11] was significant.
(7) Total physical activity:The main effect of situational regulatory focus [*F*(1, 97) = 2.15, *p* = 0.15] and the exercise information frame [*F*(1, 97) = 14.74, *p* = 0.13] were not significant. However, the interaction was significant (see Figure 4E), *F*(1, 97) = 51.27, *p* < 0.01, *η_p_*^2^ *=* 0.35.

Further simple-effects analyses showed that under the positive frame, promotion-focused participants scored significantly higher on information value [*F*(1, 97) = 16.95, *p* < 0.01, *η_p_*^2^ *=* 0.15], emotional intensity [*F*(1, 97) = 14.69, *p* < 0.01, *η_p_*^2^ *=* 0.13], behavioral intention [*F*(1, 97) = 23.23, *p* < 0.01, *η_p_*^2^ *=* 0.19], vigorous physical activity [*F*(1, 97) = 51.25, *p* < 0.01, *η_p_*^2^ *=* 0.34], and total physical activity [*F*(1, 97) = 38.23, *p* < 0.01, *η_p_*^2^ = 0.28] than those primed with a prevention focus. When faced with the negative frame, participants primed with a prevention focus scored significantly higher on emotional intensity [*F*(1, 97) = 6.20, *p* = 0.01, *η_p_*^2^ *=* 0.06], vigorous physical activity [*F*(1, 97) = 17.60, *p* < 0.01, *η_p_*^2^ *=* 0.15], and total physical activity [*F*(1, 97) = 16.06, *p* < 0.01, *η_p_*^2^ = 0.14] than those primed with a promotion focus. Put it differently, participants primed with a promotion focus scored significantly higher on information value [*F*(1, 97) = 12.30, *p* < 0.01, *η_p_*^2^ = 0.11], emotional intensity [*F*(1, 97) = 31.41, *p* < 0.01, *η_p_*^2^ = 0.24], behavioral intention [*F*(1, 97) = 3.90, *p* = 0.05, *η_p_*^2^ = 0.04], vigorous physical activity [*F*(1, 97) = 78.11, *p* < 0.01, *η_p_*^2^ = 0.44], and total physical activity [*F*(1, 97) = 61.41, *p* < 0.01, *η_p_*^2^ = 0.38] after reading the positively framed message than after reading the negatively framed message. Participants primed with a prevention focus scored higher on behavioral intention [*F*(1, 97) = 14.27, *p* < 0.01, *η_p_*^2^ = 0.13], vigorous physical activity [*F*(1, 97) = 6.25, *p* = 0.01, *η_p_*^2^ = 0.06], and total physical activity [*F*(1, 97) = 5.46, *p* = 0.02, *η_p_*^2^ = 0.05] after receiving the negative frame than after receiving the positive frame.

We divided participants into the regulatory fit group (promotion focus/positive frame group and prevention focus/negative frame group) and the regulatory non-fit group (promotion focus/negative frame group and prevention focus/positive frame group) to further verify the overall regulatory fit effect. The *t* test results showed a significant difference between the fit group and the non-fit group on all dependent variables except walking physical activity and moderate activity, as shown in Table 5.

**Table 5:** Situational regulatory fit effect in Study 2 (M ± SD).

Independent variables	Fit	Non-fit	*t*	*p*	*d*
Information value	5.44 ± 0.67	4.88 ± 0.88	3.59	0.00	0.72
Emotional intensity	5.04 ± 0.96	4.23 ± 0.95	4.25	0.00	0.85
Behavioral intention	5.21 ± 0.86	4.51 ± 0.93	3.92	0.00	0.78
Vigorous PA	2,243.14 ± 1,094.21	1,004.80 ± 505.23	7.33	0.00	1.47
Moderate PA	888.62 ± 588.94	710.40 ± 408.27	1.76	0.08	0.35
Walking PA	402.55 ± 282.97	320.50 ± 220.91	1.62	0.11	0.32
Total PA	3,534.31 ± 1,398.95	2,035.70 ± 733.67	6.72	0.00	1.35

##### 3.2.2.3 Mediation analysis: linking Fit to exercise *via* persuasive outcomes

To examine the mediating role of persuasive outcomes in the regulatory fit-physical activity relationship, we conducted mediation analyses using Model 4 of the PROCESS macro for SPSS. The analytical protocol involved: (1) operationalizing regulatory fit as a dummy-coded independent variable (Non-fit = 1, Fit = 2), (2) standardizing and aggregating three persuasive measures (information value, emotional intensity, and behavioral intention) to form a composite mediator (persuasion effect index), and (3) separately analyzing standardized vigorous physical activity (VPA) and total physical activity (TPA) scores as dependent variables. Model 1 revealed significant mediation pathways for VPA (Table 6). Regulatory fit exerted both direct [*β* = 0.51, *p* < 0.01, 95% CI (0.34, 0.67)] and indirect effects through persuasive outcomes [*β* = 0.07, *p* < 0.05, 95% CI (0.01, 0.15)]. The total effect reached statistical significance [*β* = 0.58, *p* < 0.01, 95% CI (0.43, 0.78)], indicating partial mediation of the regulatory fit-VPA relationship by persuasive outcomes. Parallel analysis for TPA in Model 2 demonstrated similar mediation patterns with attenuated effects (Table 6). We observed a significant direct effect [*β* = 0.39, *p* < 0.01, 95% CI (0.21, 0.57)] and indirect pathway through persuasion [*β* = 0.05, *p* < 0.05, 95% CI (0.01, 0.14)]. The total effect remained significant [*β* = 0.45, *p* < 0.01, 95% CI (0.27, 0.62)], confirming partial mediation in the regulatory fit-TPA association.

**Table 6:** Bootstrap test on the indirect effects of depression on relapse.

Model	Effect	Effect value	SE	LLCI	ULCI
Model 1	Direct effect	0.51	0.08	0.34	0.67
	Indirect effect	0.07	0.03	0.01	0.15
	Total effect	0.58	0.07	0.43	0.73
Model 2	Direct effect	0.39	0.09	0.21	0.57
	Indirect effect	0.05	0.04	0.01	0.14
	Total effect	0.45	0.08	0.27	0.62

LLCI, the lower limit of the confidence interval; ULCI, the upper limit of the confidence interval; Model 1, regulatory fit → persuasion effect → vigorous PA; Model 2, regulatory fit → persuasion effect → total PA. All variables in the model were standardized and then put into the regression equation.

## 4 Discussion

In this study, the regulatory fit theory was applied to explore the psychological benefits of matching regulatory focus (chronic/situational) with corresponding exercise information frame. Two experiments showed that individuals were inclined to evaluate the exercise information more positively, show higher emotional intensity, and be more willing to engage in physical activity when the exercise information frame matched with either their chronic or situational regulatory focus. More importantly, the results in Study 2 also revealed an enduring effect of regulatory fit on exercise behavior, showing regulatory fit led to more engagement in vigorous physical activity in the following week.

The current investigation advances regulatory fit theory in exercise behavior through four distinct contributions. First, the majority of current research on physical activity promotion primarily relies on external intervention methods, with limited integration of individual characteristics and advocacy information. However, it is imperative to amalgamate these two factors in research endeavors. This study investigated the impact of regulatory focus as a representation of individual characteristics and an information framework for physical activity on perceived value and participation intention under both separate and interactive mechanisms. Second, although regulatory focus can be both chronically salient and situationally activated, most studies paid attention to only one form of regulatory focus when testing the effect of regulatory fit on exercise-related variables. Our two studies found that these two forms of regulatory focus had very similar effect on exercise-related cognition, emotion, and intention. The effect sizes (partial *η*² = 0.11–0.17) were consistent with those reported in previous research on regulatory fit [ (31): *f* = 0.34–0.39 (32);: *f* = 0.34–0.36 (23);: *f* = 0.23–0.42], underscoring the well-established impact of aligning individuals' motivational orientations with message framing. Third, not only did we examine the immediate effect of regulatory fit, but we also tracked participants' actual behavior for one week and investigated the enduring effect of regulatory fit, which provides greater ecological validity. The results revealed notably stronger effects for vigorous physical activity (*η*² = 0.17) and total physical activity (*η*² = 0.35), exceeding the typical effect sizes observed in laboratory-based motor tasks. Similarly, Spiegel et al. (38) demonstrated that participants exposed to regulatory-fit-compatible messaging exhibited significantly greater behavioral improvements (e.g., a 21% increase in fruit and vegetable intake) compared to non-fit conditions over one week. These findings provide preliminary support for the translational potential of regulatory fit. However, these effects were only tested over a one-week period. Future research is needed to determine whether the benefits of regulatory fit can be sustained over longer durations. Fourth, our findings reveal robust regulatory fit effects in moderately active individuals, challenging Kay and Grimm's (23) assertion that such effects predominantly benefit exercise-naïve populations. This discrepancy stems from key methodological differences: Unlike prior studies categorizing exercise experience dichotomously (low vs. high), our focus on undergraduates with habitual yet non-elite activity patterns identified a transitional cohort retaining motivational plasticity—a population overlooked in earlier frameworks. Participants' routine exposure to physical activity in academic environments may have amplified their sensitivity to identity-aligned message framing. Crucially, our use of situational priming (rather than chronic regulatory focus measures) likely disrupted habitual tendencies, enabling fit-driven motivation even in moderately active individuals. Longitudinal tracking of vigorous physical activity, a behavior requiring conscious effort rather than routine habituation, further heightened sensitivity to regulatory fit effects. These insights underscore the need for future studies to adopt gradient-based stratification (naïve/moderate/elite) to clarify boundary conditions of regulatory fit.

It is intriguing why regulatory fit has such beneficial effect. According to the regulatory fit theory, regulatory focus acts as a “filter” that makes individuals more sensitive to corresponding information (39). When regulatory focus and the information frame achieve regulatory fit, individuals' ventromedial prefrontal cortex would be activated, which makes them more favorable to the advocacy message (40) and helps them process information more fluently (38). Furthermore, regulatory fit could also improve individuals' sense of identity and correctness (41), enhancing concentration and experience value in decision-making (42). As a result, regulatory fit is advantageous for individuals in terms of cognition, emotion, intention, and behavior. Notably, recent theoretical advancements integrate regulatory focus theory (RFT) with self-determination theory (SDT) through the Need-Support Model (NSM), offering a nuanced perspective on how regulatory fit operates (43). SDT posits that autonomy, competence, and relatedness are fundamental psychological needs that drive motivation and well-being. The NSM bridges RFT and SDT by proposing that regulatory focus interacts with need satisfaction: Promotion-focused individuals exposed to gain-framed messages (e.g., “exercise enhances immunity”) experience heightened perceptions of autonomy (“I freely choose to exercise”), competence (“I can achieve fitness goals”), and relatedness (“exercise connects me to others”). This inflated need satisfaction elevates information valuation, triggers positive emotions (e.g., excitement), and reinforces intentions by framing vigorous activity as self-actualization rather than obligation. Conversely, prevention-focused individuals receiving loss-framed messages (e.g., “inactivity increases disease risk”) mitigate threats to autonomy (“I must exercise to avoid harm”) and competence (“I can prevent risks”), thereby preserving cognitive acceptance. By maintaining relatedness through perceived social responsibilities (e.g., “exercise fulfills family duties”), external pressures are transformed into controllable goals, reducing anxiety and stabilizing behavioral intentions. This dual-pathway mechanism—enhancing need satisfaction for promotion-focused individuals and minimizing need frustration for prevention-focused individuals—explains how regulatory fit sustains motivation across cognitive, affective, and behavioral domains. Such integration underscores the NSM's value in elucidating how motivational orientations and need-supportive environments jointly shape engagement in health behaviors and beyond.

Therefore, individuals can take advantage of the regulatory fit effect when engaging in physical activity. For instance, health management professionals and policymakers can effectively target individuals' regulatory focus to provide personalized physical activity information, thereby fostering individual exercise preferences. On one hand, data analysis can be utilized to construct an individual profile and discern their more stable trait regulatory focus. On the other hand, situational factors can stimulate the state regulatory focus of individuals. To promote physical activity among individuals with a promotion focus, it is recommended to encourage or induce positive mental associations following exercise in order to establish a positive body image or state. Conversely, for individuals with a preventive orientation, they should be guided to envision the physical characteristics resulting from lack of exercise and enhance their motivation to engage in physical activities by avoiding negative images. Once the regulatory fit is achieved, it will enhance motivation and enthusiasm for exercise, increase persistence in engaging in sports behavior, and maximize the persuasive impact of sports information.

It has to be noted that this study found insignificantly weak effect of regulatory fit on moderate physical activity and walking activity. Moderate physical activity refers to activities that induce a slightly faster heartbeat, such as lifting light objects and cycling at a regular speed. Walking is defined as moving from one place to another for recreation and exercise. The insignificance of the difference in these activities between the fit and non-fit groups may be attributed to a ceiling effect, such that moderate physical exercise and walking are common activities for college students' daily lives, especially for our participants who were from a sport university.

Finally, there are some limitations in our study. First, the measurement of exercise behavior needs to be improved. Although we utilized a subjective approach to monitor exercise behavior, potential issues remain. For instance, the international physical activity questionnaire relies on subjective recall, which may be influenced by memory bias, social desirability bias, emotional motivation and other factors that could compromise the scale's reliability and validity. More accurate and portable assessments could be used in follow-up research to measure objective exercise behavior. Second, the current research did not test chronic and situational regulatory focuses simultaneously, which requires a much larger sample size to achieve adequate statistical power. It is intriguing for future research to explore which form of regulatory fit is more dominant when individuals' chronic regulatory focus and situationally activated focuses are discrepant. Third, the homogeneity of our sample (university students with habitual yet non-elite activity patterns) may constrain the ecological validity of findings. Future studies should prioritize diversified recruitment strategies and incorporate measures of sociocultural and motivational covariates to enhance generalizability.

## 5 Conclusion

When there is a fit between the exercise information frame and regulatory focus, individuals tend to evaluate the exercise information more positively, show more positive emotions, are more willing to take part in exercise, and may engage in more actual physical activity. This study broadens the scope of the regulatory fit theory's applicability, promotes the development of sports and exercise research topic, and provides guidance for the practice of physical activity.

## Data Availability

The datasets presented in this study can be found in online repositories. The names of the repository/repositories and accession number(s) can be found below: https://osf.io/vj2gm/?view_only=a12a3b0df1be4e73981d92bde5f13d26.
