# Supplementary material for: Promoting physical activity: the contribution of regulatory fit
Source: Front Sports Act Living. 2025 May 14;7:1564917. doi: 10.3389/fspor.2025.1564917 (PMC12116541; doi:10.3389/fspor.2025.1564917)
Supplement: Supplementary file 1 [file Table1.docx]

**Regulatory Focus Questionnaire**

The following questions describe specific occurrences in your life. Please select the proper answer based on the frequency with which the occurrences occur. 1 = never, 2 = rarely, 3 = occasionally, 4 = often, and 5 = always for questions 1 through 8. For questions 9 and 10, 1 = completely incorrect, 2 = incorrect, 3 = uncertain, 4 = correct, and 5 = completely accurate.

|  | never | rarely | occasionally | often | always |
| --- | --- | --- | --- | --- | --- |
| 1. Relative to the majority of individuals, you rarely achieve your life goals? | 1 | 2 | 3 | 4 | 5 |
| 2. When you were a child, did you frequently do things that your parents could not tolerate? | 1 | 2 | 3 | 4 | 5 |
| 3. you have accomplished something; does this success motivate you to try harder? | 1 | 2 | 3 | 4 | 5 |
| 4. Did you frequently irritate your parents as you were growing up? | 1 | 2 | 3 | 4 | 5 |
| 5. While you were growing up, did you frequently do things that your parents deemed inappropriate? | 1 | 2 | 3 | 4 | 5 |
| 6. When I pursue something I deem significant, I discover that I do not do as well as I would like. | 1 | 2 | 3 | 4 | 5 |
| 7. Do you frequently perform well at the numerous activities you desire to do? | 1 | 2 | 3 | 4 | 5 |
| 8. Do you adhere to your parents' regulations at all times? | 1 | 2 | 3 | 4 | 5 |
|  | completely incorrect | incorrect | uncertain | correct | completely accurate |
| 9.I believe I have made progress toward achievement. | 1 | 2 | 3 | 4 | 5 |
| 10. I have few hobbies or activities that fascinate me or keep me completely occupied. | 1 | 2 | 3 | 4 | 5 |

**Persuasion Effect Questionnaire**

Please carefully read the following information and respond to the subsequent questions.

|  | Extremely inconsistent | Inconsistent | Slightly inconsistent | Consistent | Extremely consistent |
| --- | --- | --- | --- | --- | --- |
| 1. This information is highly authoritative. | 1 | 2 | 3 | 4 | 5 |
| 2. This information is quite reliable. | 1 | 2 | 3 | 4 | 5 |
| 3. This information is incredibly persuasive | 1 | 2 | 3 | 4 | 5 |
| 4. I am quite pleased after reading the message. | 1 | 2 | 3 | 4 | 5 |
| 5. I'm saddened by this message's contents. | 1 | 2 | 3 | 4 | 5 |
| 6. After reading this, I became interested in learning more about the effects of physical activity. | 1 | 2 | 3 | 4 | 5 |
| 7. After reading this information, I will make an effort to exercise in the future. | 1 | 2 | 3 | 4 | 5 |

**Situational Regulatory Focus Priming Material**

**Promotion focus**

**1. The self-directed task**

Everyone has hopes, dreams, and goals in life (i.e., those things we want to pursue or people we want to be). Please think of two hopes or goals you've had in the past and two you have now, and write them down.

Previous desires:

① ② Current desires:

① ②

**2. The maze task on paper**

A piece of Swiss cheese is placed at the maze's exit, while the mouse is located in the maze's center. Please sketch a path to help the mouse escape the maze and eat the Swiss cheese.


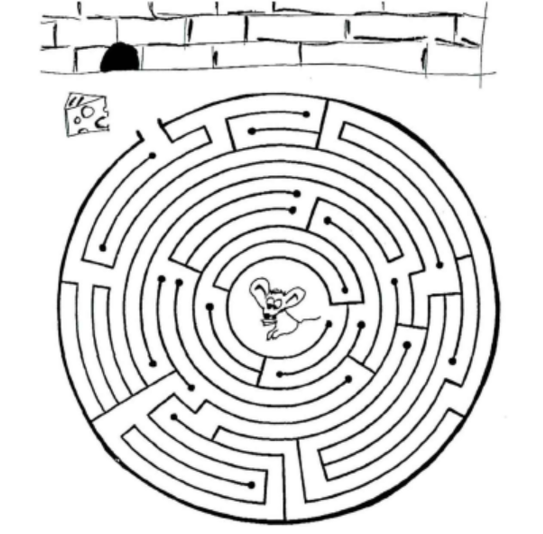


**Prevention focus**

**1. The self-directed task**

Everyone has duties, responsibilities, and obligations in his or her lifetime (i.e., those we feel we have to do, such as study, getting a job, caring for a sick parent, etc.). Please recollect and mention two of your past and present responsibilities and obligations.

Previous responsibilities:

① ② Current responsibilities:

① ②

**2. The maze task on paper**

A hungry owl hovered over the maze, preparing to pounce on the mouse in the center and devour it. Please create a path for the rats to escape the maze and avoid the owls.


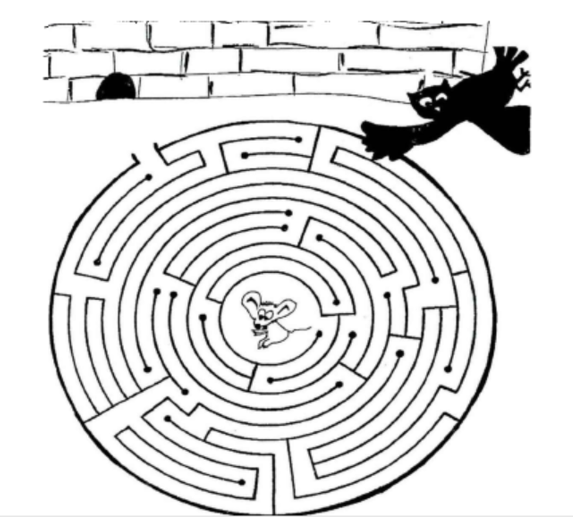


**Manipulation Check Questionnaire**

| 1. I prefer to act in accordance with the consensus. | 1 | 2 | 3 | 4 | 5 | 6 | 7 | I prefer to pursue my own interests. |
| --- | --- | --- | --- | --- | --- | --- | --- | --- |
| 2. I favor repayment of my debt. | 1 | 2 | 3 | 4 | 5 | 6 | 7 | I prefer to travel internationally. |
| 3. I am willing to do whatever it takes to accomplish my obligation. | 1 | 2 | 3 | 4 | 5 | 6 | 7 | I would rather travel wherever my heart directs me. |

**Manipulation of the information frame**

1. Positive Frame (Gain-Framed):
Regular physical activity is scientifically proven to bring substantial health benefits. By engaging in moderate exercise (e.g., brisk walking, cycling) for at least 30 minutes daily, you can enhance metabolic efficiency, helping your body burn calories more effectively. Over time, this may improve physical fitness and muscle strength, leading to a toned and healthy physique. Additionally, consistent exercise strengthens immune system function, reducing susceptibility to infections, and lowers the risk of chronic diseases. Start exercising today to actively invest in your long-term well-being!

2. Negative Frame (Loss-Framed):
Scientific evidence shows that insufficient physical activity may lead to serious health consequences. If you fail to engage in moderate exercise (e.g., brisk walking, cycling) for at least 30 minutes daily, you might experience reduced metabolic efficiency, making it harder to maintain a healthy weight. Prolonged inactivity can also contribute to loss of muscle mass and physical stamina, resulting in a weaker body image. Furthermore, it may compromise immune system function, increasing vulnerability to infections, and raise the risk of developing chronic diseases. Avoid these negative outcomes by prioritising exercise in your daily routine.

**International Physical Activity Questionnaire**

1. During the last 7 days, on how many days did you do vigorous physical activities like heavy lifting，digging，aerobic exercise or cycling rapidly? Think about only those physical activities that you did for at least 10 minutes at a time.

days per week

No vigorous physical activity Skip to question 3.

2. How much time did you usually spend on one of those days doing vigorous physical activities?

hours per day

minutes per day

3. Again，think about only those physical activities that you did for at least 10 minutes at a time. During the last 7 days, on how many days did you do moderate physical activities like carrying light loads, cycling at a moderate pace, or double table tennis? Please do not include walking.

days per week

No moderate physical activity skip to question 5.

4. How much time did you usually spend on one of those days doing moderate physical activities ?

hours per day

minutes per day

5. During the last 7 days, on how many days did you walk for at least 10 minutes at a time like walking from place to place for amusement, sport, exercise, and recreation?

days per week

No walking skip to question 7.

6. How much time did you usually spend on one of those days walking?

hours per day

minutes per day

7. During the last 7 days, on how many days did you keep sedentary?

hours per day

minutes per day
